# Adenomatoid tumors of the testis: A report of two cases and review of the literature

**DOI:** 10.3892/ol.2014.1938

**Published:** 2014-03-05

**Authors:** DUQUN CHEN, ZUHU YU, LIANGCHAO NI, YAOTING GUI, SHANGQI YANG, BENTAO SHI, YONGQING LAI

**Affiliations:** 1 Department of Urology, Peking University Shenzhen Hospital, Shenzhen PKU-HKUST Medical Center, Shenzhen 518036, P.R. China; 2 The Guangdong and Shenzhen Key Laboratory of Male Reproductive Medicine and Genetics, Institute of Urology, Peking University Shenzhen Hospital, Shenzhen PKU-HKUST Medical Center, Shenzhen 518036, P.R. China; 3 Anhui Medical University, Hefei, Anhui 230032, P.R. China

**Keywords:** adenomatoid tumors, asymptomatic mass, scrotal neoplasms

## Abstract

Adenomatoid tumors are rare benign neoplasms that normally occur in the scrotum. The clinical symptoms and routine examinations mean that it is difficult to distinguish adenomatoid tumors from malignant intratesticular solid tumors, which may result in unnecessary orchidectomies. The present report describes two adenomatoid tumor patients treated between 2006 and 2013 at the Peking University Shenzhen Hospital who presented with an asymptomatic mass in the scrotum. Based on thorough analysis of clinical features, blood, radiological images and intra-operative findings, limited local excisions were performed, revealing adenomatoid tumors of the testis on pathological examination. The patients were followed up and exhibit no recurrence at the time of writing. The present report also summarizes the morphological and immunohistochemical features of paratesticular tumors and reviews the literature to improve understanding of these rare lesions and assist in accurate diagnosis.

## Introduction

Adenomatoid tumors are benign masses usually located within the genital tract organs of males and females (1). The majority involve the epididymis, but sites may also include the testicular tunica, spermatic cord and, in rare cases, the testicular parenchyma (2–4). Extragenital sites of involvement include the heart, lymph nodes, adrenal glands, intestinal mesentery, omentum and retroperitoneum (5). The majority present as small, firm, asymptomatic intrascrotal masses which can be mistaken for a large variety of diseases, including benign lesions and intratesticular malignancies. In numerous cases, diagnosis of adenomatoid tumors results from incidental pathological findings following orchidectomy. Therefore, it is imperative that urologists be aware of the features of this rare lesion to avoid erroneous diagnosis and unnecessary extensive surgical excision. The patients provided their written informed consent.

## Case reports

Between 2006 and 2013, two patients with a one-year history of an asymptomatic mass in the scrotum (Table I) were admitted to Peking University Shenzhen Hospital (Shenzhen, China).

### Case 1

A 40-year-old patient presented with a palpable and painless swelling with a gradual increase in testicular size over one year. The patient had no history of local trauma or surgery to the scrotum. Local examination revealed a firm and painless mass of 1×1 cm fixed to the testis in the left side of the scrotum. The overlying skin appeared normal and the inguinal lymph nodes were not palpable. Serum levels of α-fetoprotein (AFP) and β-human chorionic gonadotrphin (β-HCG) were within normal range. Ultrasonography revealed a well-defined, heterogeneous, round nodule with no capsule at the lower pole of the testis. Computed tomography scan of the pelvis revealed no significant intra-abdominal lymphadenopathy.

Under suspicion of malignancy, radical orchidectomy was prescribed. During the surgery, paratesticular nodular masses were identified between the tunica vaginalis and the head of the epididymis, firm in consistency with no evidence of invasion which is indicates that there is a high possibility of a benign lesion.

### Case two

A 39-year-old male presented with a palpable mass in the right side of the scrotum. There was no history suggestive of urinary infection, genito-urinary tuberculosis or epididymo-orchitis. On local examination, a mass was identified in the right testis which was 2×1.5 cm in size, firm in consistency and well-marginated with smooth swelling. The overlying skin and surrounding adenexa appeared normal. Serum levels of AFP and β-HCG were within normal limits. Scrotal ultrasound revealed a solid mass in the epididymal body of the right scrotum that was hypoechoic compared with the testicular parenchyma.

### Management

**Figure 1:**
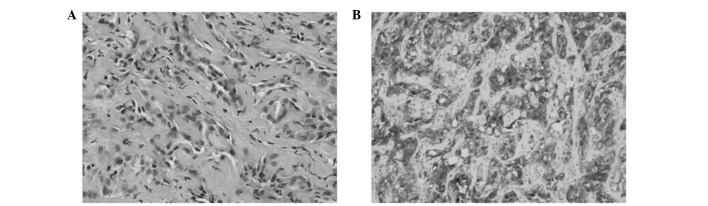
Adenomatoid tumor of the testis. (A) Hematoxylin-eosin stain revealing tubules of cuboidal cells with occasional cytoplasmic vacuolation. Nuclei are bland with dispersed chromatin and surrounded by fibrous stroma (magnification, ×200). (B) Immunohistochemical staining revealing marked Wilms’ tumor gene 1 expression by neoplastic cells (magnification, ×100).

Limited local excisions were performed in the two cases and the morphological and immunohistochemical features are presented in Table II and Fig. 1. At the time of writing, follow-ups have revealed no recurrence.

## Discussion

Adenomatoid tumors are relatively uncommon benign tumors of mesothelial origin frequently located within the genital tract organs (6). In the female genital tract, adenomatoid tumors may be located in the uterus, fallopian tubes and ovarian hilus, and the epididymis, spermatic cord, prostate and ejaculatory duct in the male (1). Adenomatoid tumors account for ~30% of all paratesticular tumors and are are therefore the most common type of paratesticular tissues (7). In contrast to testicular neoplasms, which are 95% malignant, paratesticular tumors are more frequently benign and may be cured by local excision (7). However, it is not always easy to differentiate between the two masses.

Adenomatoid tumors present either as an incidental finding or a slow growing scrotal mass, most often in patients between 30 and 50 years of age. Enlargement is usually painless and the scrotal skin and surrounding adenexa remain normal. The majority of adenomatoid tumors are present asymptomatically for several years and are uniformly benign. Rarely, patients have presented with testicular pain. In the majority of cases*,* adenomatoid tumors present as painless, firm, intrascrotal masses of <2 cm diameter.

The ultrasound features of adenomatoid tumors may vary but usually include a well-defined, homogeneous, round nodule with variable echogenicity ranging from hypoechoic to hyperechoic. On magnetic resonance imaging, such masses are slightly hypointense compared with the testicular parenchyma on T2-weighted images and, on post-contrast images, exhibit similar enhancement to the testis (8).

Microscopically, they contain a number of histological patterns: adenoid or tubular glandular, angiomatoid, solid, cystic or transitional (9), which may pose a range of diagnostic problems, particularly for differentiating adenomatoid tumors from other paratesticular tumors (10). An understanding of the pathological features of paratesticular tumors is crucial for accurate diagnosis (Table III) (9,10).

An asymptomatic mass in the scrotum is a nonspecific symptom that can be caused by benign lesions and malignances. Ultrasound has become a first-line imaging technique, used to assess acute and nonacute conditions of the scrotum, and in many cases it is the only modality required (8). When ultrasound confirms that the mass is extratesticular (particularly if located in the epididymis), circumscribed and homogeneous with no evidence of invasion to adjacent tissue, in addition to typical clinical features and normal levels of serum tumor makers, a diagnosis of paratesticular mass can be made, as in case two.

In many cases, the similar imaging studies and clinical signs make it hard to distinguish adenomatoid tumors from malignant intratesticular tumors, leading to unnecessary extensive surgical excision of the whole testis (11). The present study is the first reported case to avoid extensive surgical excision based on intra-operative evaluation when this dilemma was encountered.

In summary, adenomatoid tumors in the testis are rare disorders that may pose difficulty for diagnosis, particularly when occurring in unusual locations where the aim is to preserve endogenous testicular function. This report presents two cases of adenomatoid tumors of the testis with the symptom of asymptomatic mass in the scrotum. Local excisions were performed based on thorough analysis of the clinical features, tumor makers, radiological images and intra-operative findings. A complete understanding of the pathological features of paratesticular tumors is critical in making accurate diagnoses.

## Figures and Tables

**Table I:** Physical characteristics and clinical and radiological features of the patients.

No.	Age, years	Chief complaint	Date of presentation	AFP	β-HCG	Ultrasonography	CT
1	40	Asymptomatic mass in scrotum	2006	Normal	Normal	Well-defined heterogeneous nodule	No metastasis
2	39	Asymptomatic mass in scrotum	2013	Normal	Normal	Hypoechoic homogeneous mass	Not performed

AFP, α-fetoprotein; β-HCG, β-human chorionic gonadotrophin; CT, computed tomography.

**Table II:** Morphological and pathological features of the masses.

No.	Size, cm	Density	Location	Microscopic features	Immunohistochemistry
1	1×1	Firm	Tunica vaginalis	Channels lined by flattened cells with cytoplasmic vacuolation, myxoid change	CK^+^, D2–40^+^, CR^+^, CD68^−^
2	2×1.5	Firm	Epididiymis	Tubules of cuboidal cells with cytoplasmic vacuolation, bland nuclei, fibrous stroma	WT1^+^, calretinin^+^, D2–40^+^, CK5/6^−^

CK, cytokine; CR, complement receptor; CD, cluster of differentiation; WT1, Wilms’ tumor gene 1.

**Table III:** Morphological and immunohistochemical features of paratesticular tumors.

Lesion	Microscopic features	Immunohistochemistry
Adenomatoid tumor	Cords and tubules of cuboidal to columnar cells with vacuolated cytoplasm and fibrous stroma	WT1^+^, D2–40^+^, calretinin^+^, CK5^+^, vimentin^+^
Fibrous pseudotumor	Dense fibrous tissue with interspersed fibroblasts and mixed inflammatory cells	Not clear
Cystadenoma	Epithelial-like tumor cells with a sertoliform growth pattern and cystic dilatations	Inhibin^+^, S-100^+^, CD99^+^
Leiomyomas	Interlacing and whorling bundles of smooth muscle cells	Desmin^+^
Serous borderline tumor	Cystic with numerous intracystic, blunt papillae lined by stratified epithelial cells having minimal to mild cytologic atypia	ER^+^, PR^+^, CD15^+^, MOC-31^+^, calretinin^−^
Mesothelioma	Epithelioid cells arising from the tunica vaginalis with papillary, tubulopapillary or solid architectural patterns	Calretinin^+^, WT1^+^, D2–40^+^, CK7^+^, CK5/6^+^

WT1, Wilms’ tumor gene 1; CK, cytokine; CD, cluster of differentiation; ER, estrogen receptor; PR, progesterone receptor.
